# MeV Ion Beam Analysis

**DOI:** 10.6028/jres.093.123

**Published:** 1988-06-01

**Authors:** J. A. Cookson, T. W. Conlon

**Affiliations:** Harwell Laboratory, UKAEA Oxfordshire OX11 ORA, UK

## 1. Introduction

The main techniques traditionally used in MeV Ion Beam Analysis are Particle Induced X-ray Emission (PIXE), Rutherford Backscattering (RBS) and Nuclear Reaction Analysis (NRA), which, broadly speaking, utilize light (*m*⩽4) ions. These techniques can also be applied in the channeling mode to study the structural properties of crystalline materials (such as foreign atom location, radiation damage, and interface studies). The techniques have the common feature that all are based on processes that occur spontaneously in the interaction of light ion beams with solids. Since these processes are well understood and are very well established in the literature [1–3], this review will treat them only with regard to improvements in their quantification (sec. 3).

Heavy ion beams in solids exhibit additional properties that can be utilized in materials. For example, they transfer large amounts of energy in collision with other nuclei; they slow down rapidly and in doing so generate regions of very high ionization density. Analytical methods based on the generation of fast recoils, on the nuclear physics process of coulomb excitation and on the heavy ion induced desorption of atoms and molecules are under development and are described in section 2.

Both the light and heavy ion techniques can be applied either in the broad beam or microbeam mode. New developments are taking place in the production and focusing of micrometer size beams for analysis where lateral resolution is important. These advances, which could revolutionize the prospects for microbeam systems, are described in section 4.

## 2. Advanced Analysis Techniques

The power of MeV ion beam analysis can be extended in several ways: by the use of heavy ions, by measuring more than one parameter of the interaction process, and by the application of coincidence techniques. Some examples are given below.

### 2.1 ERDA

The Elastic Recoil technique (ERDA) utilizes fast heavy ions to produce recoil substrate atoms that are ejected from the substrate and can be subsequently analyzed. In contrast to the light ion techniques that generally provide only one parameter (usually a particle or proton energy), both recoil mass and recoil energy can be measured.

**Figure 1:**
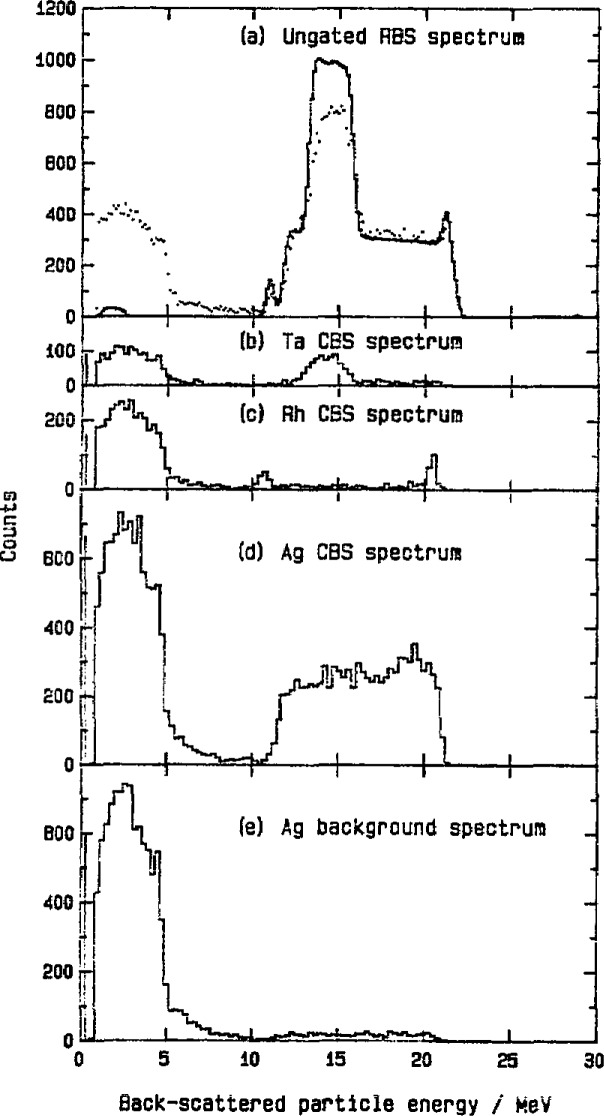
A one-parameter ERDA measurement with 30 MeV ^35^Cl ions incident on an amorphous Si solar cell produced by plasma decomposition of silane and B_2_H_4_. The solid line is a simulation assuming uniformity of H and B concentration with depth. A surface C peak appears near channel 570 [4].

ERDA is in routine use as a single parameter technique, where an energy spectrum of all the ions recoiling from the specimen can be interpreted to give depth profiles of the various elements (see fig. 1). There are several ways of developing a two-parameter version of ERDA so that a separate energy spectrum is produced for each type of recoiling ion. One technique carries out mass spectrometry of the ions with electric or magnetic fields. Another possibility is the application of the stopping power equation *E*d*E*/d*x* = *kMQ*^2^ where d*E* is measured in a thin detector and the total energy *E* in a thick detector. *M* and *Q* are the ion mass and charge.

Also used is the time-of-flight (TOF) technique in which the velocity *V* of the ion is obtained from its transit time over a fixed distance, so that, if the ion energy $\left(\frac{1}{2}MV^{2}\right)$ is also measured, the mass *M* can be uniquely determined.

All of these options, extensively used in nuclear physics experiments, are being investigated in various laboratories world-wide for material science uses [2,3]. However, no detailed comparison of the respective merits of the various options has yet been published.

### 2.2 Coulomb Excitation Studies

RBS has proved extremely useful in characterizing heavy element distributions in light element substrates but, because it measures only a single parameter (i.e., *E*) and because of the small change in mass-energy product between neighboring elements, it is unable to resolve distributions of elements that are similar in mass (e.g., P in Si, Ga in As compounds). ERDA is very useful for determining multi-light element depth distributions, especially with two-parameter measurement of *M* and *E* (as indicated above). However, unless very heavy high energy ions are used, the recoil energies imparted to elements such as P, Si, Ga and As are insufficient to enable them to escape from the substrate.

Coulomb excitation in coincidence with RBS (e.g., called CBS) offers a solution to this class of problem. In this case the two-parameter measurement of RBS energy and gamma-ray energy from the recoiling nuclei is sufficient to distinguish the depth profiles of different nuclides even if their mass is the same. This is because each nuclide has a unique gamma-ray signature.

**Figure 2:**
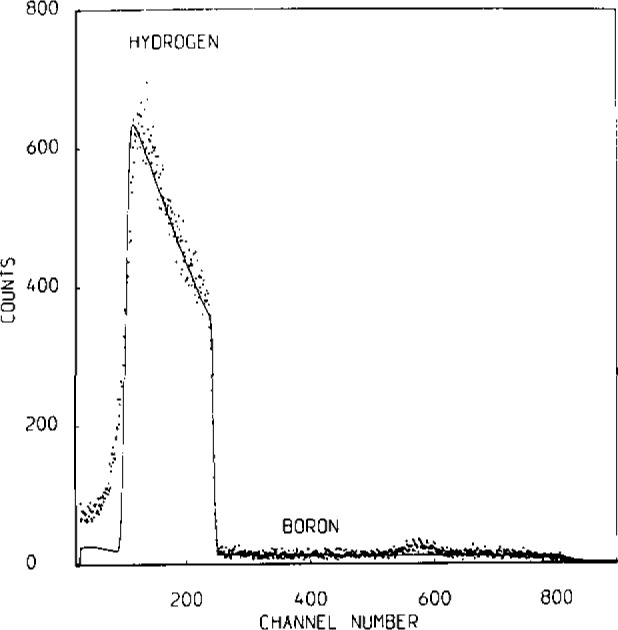
Spectra from the analysis of a composite target consisting of thin foils of Al, Ag, Rh, and Ta (with the Al at the front). The beam is 40 MeV ^16^O and RBS is carried out at 170°. The CBS spectra are obtained by gating the RBS by characteristic gamma-rays from the various nuclides [5].

Figure 2 shows a preliminary test of the method, but insufficient data is yet available to determine the limits of depth resolution and sensitivity.

Bakir et al. [6] have reported an earlier attempt to combine RBS and PIXE (i.e., utilizing x-rays rather than the gamma-rays from Coulomb Excitation discussed above). They used 34 MeV ^16^O ions and grazing angle RBS to demonstrate a depth resolution of 70 Å and a sensitivity of 10^16^ atoms/cm^2^ from medium mass elements (Cu on Ge substrate).

### 2.3 Particle Desorption Mass Spectrometry (PDMS)

The high ionization density of both fission fragments and accelerated heavy ions has been utilized to investigate the ion induced desorption of atomic and molecular species from surfaces. Some species are often detected by TOF spectrometry as discussed above for ERDA. The phenomenon was first observed by MacFarlane and Torgerson [7] and used for the characterization of large, volatile biomolecules.

Most recently Schweikert et al. [8] have investigated the prospects of developing a technique based on this process for surface analysis. In contrast to the routinely used techniques, which require fluences of typically 10^12^–10^15^ ions/cm^2^ for analysis (and appreciably more for the coincidence techniques), useful information can be obtained from PDMS with much lower fluences (especially when fission fragments are used). The detection limit was 100 μg/g for lithium in glass, and microscopic analysis on areas as small as 11 μm diameter was demonstrated. To provide depth information, the technique must be combined with surface erosion.

## 3. Advances in IBA Microbeams

There are now upwards of 30 groups worldwide regularly using the various IBA techniques with finely focused or collimated beams to measure the lateral elemental distributions of a very wide variety of materials [9,10]. The present state-of-the-art beam quality is the 100 pA of beam in a spot with FWHM of 1 μm beam achieved at Oxford and Melbourne. These groups mainly use PIXE, which has the highest cross sections of the IBA techniques, to perform trace element analysis of biological and medical thin specimens with 1 μm resolution. All the other IBA techniques are used for a very wide range of applications, but usually at lower positional resolution.

At present the highest resolution microbeams use Van de Graaff accelerators with standard ion sources to illuminate an aperture which acts as the object for a single, demagnifying magnetic quadrupole lens. Although attempts [9] are being made to improve the focusing lens, it seems likely that significant reductions of the spot size below 1 μm, or major increases in the current density at the present spot size, will require improvement of the brightness (defined roughly as current per unit area and solid angle) of the accelerator beam.

A simplified view of the factors involved indicates that reduction of the spot diameter by a factor 10 without loss of current could be achieved fairly simply if there were an increase in beam brightness of 10^4^. This implies that if the extra brightness of a field ionization source could be utilized, a microbeam size of 0.1 μm should be possible.

The type of field ionization source developed for low energy scanning transmission microscopy [11] has an intrinsic brightness about 10^6^ greater than the sources used in Van de Graaff accelerators. Legge et al. have demonstrated [12] such a source giving 21 nA at 4×10^5^ greater brightness than an RF source, and have discussed [13] how the brightness might be maintained through an MeV accelerator.

An adaptation of the field ion source by Böhringer et al. [14] uses a small pimple formed on a tungsten tip to give even brighter beams, but with only a few hours lifetime so far.

**Figure 3:**
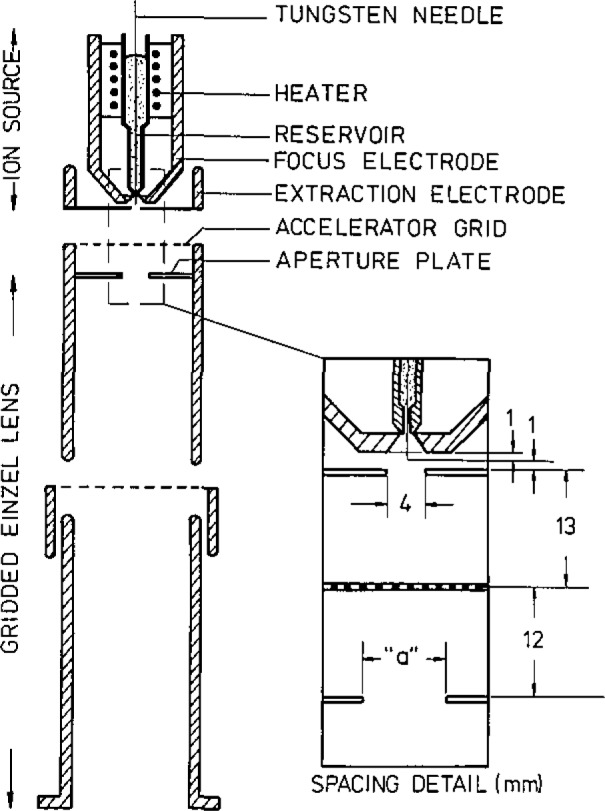
A schematic drawing of a liquid metal ion source and a gridded focusing lens [17]. A more sophisticated lens design would be needed for MeV microbeam applications.

Liquid metal sources [15] can give ion brightnesses comparable with those achieved in hydrogen field ionization source, and have the advantage that longer source life can be anticipated as the emission is from a continuously renewed “Taylor Cone” of liquid metal. So far, a gallium source has been shown [16] to work satisfactorily in a Van de Graaff accelerator at Harwell for non-microbeam use. For microbeam purposes the chosen metal is lithium which is very suitable for RBS work and also has NRA and PIXE uses. Indications are that a very simple lithium ion source system of the type shown in figure 3 would provide an appreciably brighter beam than a normal Van de Graaff ion source, but considerable development is still needed to retain that brightness as far as the microbeam object aperture.

## 4. Improved Quantification

A feature that makes all four of the main IBA techniques very different from most other analytical techniques is that their concentration and depth information are very little influenced by the chemical form of the specimen. This lets them contribute only stoichiometric information for chemical characterization, but it has the very beneficial effect of making their results almost independent of matrix considerations.

*The PIXE technique* [1] has had its widest application as a very efficient means of analyzing pollution-covered thin filters. An intercomparison of PIXE measurements with a wide range of other techniques has been described by Bombelka et al. [18] who conclude that for most contaminants giving adequate counting statistics the PIXE accuracy is better than 10%, although for S, with the lowest energy x-rays considered, allowance was needed for the filter thickness.

The rapid measurement capability of the PIXE technique is now becoming routinely applied to thick targets. These measurements are more difficult than for thin targets because the x-rays are produced at different energies and depths as the beam penetrates the specimen material and there is attenuation of the x-rays as they escape from the specimen. These introduce extra uncertainties [19] in the measurement, of which the main ones arise from the x-ray attenuation coefficients and to a lesser extent the energy dependence of the x-ray production. The analysis needs to contain some iteration as the yield of each element depends on the presence of the others. An extra aspect of this interdependence is the fluorescent production of x-rays that can occur as PIXE x-rays are absorbed in the matrix.

For biological materials, where x-ray attenuation is not very severe, PIXE measurements of IAEA H-8 Horse Kidney and NBS 1572 Citrus Leaves have been made by Clayton [20], who shows that for elements with *Z* above 18 the concentration values assumed for H, C, N and O, which are of course not measured in PIXE, affect the analysis very little. The agreement of these PIXE results with the certified values is generally at the few percent level.

Geological materials usually present a more severe challenge because of the greater influence x-ray attenuation has on the measured intensities. Nevertheless Rogers et al. [21] have shown that with sophisticated data processing, very satisfactory results can be achieved for geological standard materials.

*The RBS technique* normally uses ^4^He ions of about 2 MeV to measure depth profiles. For all except the very lightest target nuclei, the cross sections can be calculated to better than 1% accuracy provided that a screening correction [22] is applied to the Rutherford scattering law. The main extra information that is needed to interpret the RBS spectra is the way in which the incident and scattered particles lose energy as they travel through the specimen. There is now a comprehensive set of stopping power values available [23] that is believed to be accurate for ^4^He ions in most pure solid elements to better than 5%. The stopping powers for compounds are usually calculated using the Bragg Rule, but deviations of a few percent from this are believed to occur for low energy beams due to atomic binding effects.

Although the accurate knowledge of the scattering cross section does, in principle, allow RBS to be independent of standards, most practitioners find it more convenient to normalize their results to well-characterized standards. The best known of these are a series of Bi implants in silicon known as Harwell Series I, II and III, and well-characterized Ta films produced at the École Normale Supérieure in Paris. Accuracy of about ±1% is suggested [24] for the Harwell series I implants, but larger uncertainties occur among the Series II specimens [25]. RBS measurements of quantities of material can be better than ±5%, making use of such standards. The accuracy of RBS depth measurements can approach how well we know the stopping power of the ions used, i.e., perhaps 5%, and does not suffer from the uncertainties in sputtering rates that plague many depth profiling techniques.

*ERDA*, like RBS, relies on stopping power data and Rutherford cross sections. The stopping powers usually are the main source of uncertainty because, not only are the values for heavy ions less well known (typically ±10%) than those involved in RBS, but they have greater effects on the observed spectra. Each element measured in ERDA involves a different type of ion escaping from the target, and then passing through an absorber foil and a detector dead layer, before giving an energy signal. Nevertheless, accuracies of quantification and depth scale at the ±10% level can be regularly achieved. Better stopping power data would allow improvement of ERDA accuracy.

The cross sections for *NRA* do not have any simple variation with projectile or energy or from element to element, and most NRA measurements rely on comparisons with standards. Stopping powers are needed to relate results from the specimen to those from the standards, and are of course particularly important in NRA depth profiling.

An interesting indication of the accuracies that can be achieved in IBA is given by an intercomparison of three of the techniques carried out by six laboratories [26]. The two specimens consisted of nominal thickness of 30 and 100 nm of tantalum pentoxide grown anodically on Ta foil. Table 1 shows agreements among the measurements at better than the 5% level although the different groups have treated even the same techniques in different ways and relied upon different cross-section and stopping power information.

This work forms part of the Underlying Research programme of the UKAEA.

## Figures and Tables

**Table 1:** A summary of the results of an intercomparison by Seah et al. [26], using NRA, RBS and ERDA, of the thickness and composition of 30 nm and 100 nm Ta_2_O_5_ films

Technique	Laboratory	Oxygen thickness, 10^21^ atoms/m^2^		Ratio	Tantalum thickness, 10^21^ atoms/m^2^	
		30 nm	100 nm		30 nm	100 nm
NRA	Liège	1.84±0.25	5.51 ±0.74	0.333±0.003		
NRA	Compiègne	1.73±0.05	5.12±0.15	0.337 ±0.001		
NRA	Paris	1.75±0.03	5.24±0.08	0.334±0.002		
NRA	Chalk River	1.80±0.06	5.37±0.17	0.336±0.003		
RBS	Compiègne					2.08±0.07
RBS	Surrey			0.326±0.003	0.71±0.02	2.18±0.05
RBS	Harwell			0.325±0.007	0.68±0.05	2.08±0.15
ERDA	Harwell	1.73±0.14	5.64±0.45	0.307±0.026		

Average		1.77±0.05	5.38±0.21	0.328±0.010	0.70±0.02	2.11 ±0.06
